# Birth preparedness and complication readiness among the women beneficiaries of selected rural primary health centers of Dakshina Kannada district, Karnataka, India

**DOI:** 10.1371/journal.pone.0183739

**Published:** 2017-08-24

**Authors:** Kibballi Madhukeshwar Akshaya, Siddharudha Shivalli

**Affiliations:** 1 Department of Community Medicine, Yenepoya Medical College, Yenepoya University, Mangaluru, Karnataka, India; 2 Department of Public Health, Yenepoya Medical College, Yenepoya University, Mangaluru, Karnataka, India; 3 Non-Communicable Diseases Regional Technical Advisor, Southeast Asia Regional Office (SEARO), TEPHINET, A Program of the Task Force for Global Health, Inc., Decatur, Georgia, United States of America; University of Bedfordshire, UNITED KINGDOM

## Abstract

**Introduction:**

Birth preparedness and complication readiness (BPCR) is a strategy to promote timely use of skilled maternal and neonatal care during childbirth. According to World Health Organization, BPCR should be a key component of focused antenatal care. *Dakshina Kannada*, a coastal district of Karnataka state, is categorized as a high-performing district (institutional delivery rate >25%) under the National Rural Health Mission. However, a substantial proportion of women in the district experience complications during pregnancy (58.3%), childbirth (45.7%), and postnatal (17.4%) period. There is a paucity of data on BPCR practice and the factors associated with it in the district. Exploring this would be of great use in the evidence-based fine-tuning of ongoing maternal and child health interventions.

**Objective:**

To assess BPCR practice and the factors associated with it among the beneficiaries of two rural Primary Health Centers (PHCs) of *Dakshina Kannada* district, Karnataka, India.

**Methods:**

A facility-based cross-sectional study was conducted among 217 pregnant (>28 weeks of gestation) and recently delivered (in the last 6 months) women in two randomly selected PHCs from June -September 2013. Exit interviews were conducted using a pre-designed semi-structured interview schedule. Information regarding socio-demographic profile, obstetric variables, and knowledge of key danger signs was collected. BPCR included information on five key components: identified the place of delivery, saved money to pay for expenses, mode of transport identified, identified a birth companion, and arranged a blood donor if the need arises. In this study, a woman who recalled at least two key danger signs in each of the three phases, i.e., pregnancy, childbirth, and postpartum (total six) was considered as knowledgeable on key danger signs. Optimal BPCR practice was defined as following at least three out of five key components of BPCR.

**Outcome measures:**

Proportion, Odds ratio, and adjusted Odds ratio (adj OR) for optimal BPCR practice.

**Results:**

A total of 184 women completed the exit interview (mean age: 26.9±3.9 years). Optimal BPCR practice was observed in 79.3% (95% CI: 73.5–85.2%) of the women. Multivariate logistic regression revealed that age >26 years (adj OR = 2.97; 95%CI: 1.15–7.7), economic status of above poverty line (adj OR = 4.3; 95%CI: 1.12–16.5), awareness of minimum two key danger signs in each of the three phases, i.e., pregnancy, childbirth, and postpartum (adj OR = 3.98; 95%CI: 1.4–11.1), preference to private health sector for antenatal care/delivery (adj OR = 2.9; 95%CI: 1.1–8.01), and woman’s discussion about the BPCR with her family members (adj OR = 3.4; 95%CI: 1.1–10.4) as the significant factors associated with optimal BPCR practice.

**Conclusion:**

In this study population, BPCR practice was better than other studies reported from India. Healthcare workers at the grassroots should be encouraged to involve women’s family members while explaining BPCR and key danger signs with a special emphasis on young (<26 years) and economically poor women. Ensuring a reinforcing discussion between woman and her family members may further enhance the BPCR practice.

## Introduction

Maternal health is central to the development of any country to achieve equity, reduce poverty, and build social capital [1]. Reducing maternal mortality was one of the key indicators of the Millennium Development Goals [2]. Almost all the maternal deaths (99%) occur in developing countries and one-third of them occur in South Asia [3]. Birth preparedness and complication readiness (BPCR) is one of the key interventions to reduce the maternal mortality. BPCR is defined as a programmatic approach to improve the use and effectiveness of key maternal and newborn health services, based on the premise that preparing for birth and being ready for complications reduces all three phases of delays in receiving the services (i.e., delays in seeking care, reaching the healthcare facility, and in receiving adequate care at the point of service) [4]. Since a wide range of factors contribute to these delays, it requires people at multiple levels—women and their families, communities, providers, facilities, and policymakers—to engage in BPCR actions [4].

The World Health Organization (WHO) recommends that pregnant woman should receive focused ‘antenatal care’ (ANC) in which BPCR is a key component [5]. Different groups implementing safe motherhood programs have proposed various concepts of BPCR application. However, there is no single agreed-upon definition [4]. A birth plan/emergency preparedness plan includes identification of the following: knowledge of key danger signs; desired place of birth; preferred birth attendant; location of the closest appropriate healthcare facility; funds for birth-related and emergency expenses; a birth companion; transport to a health facility for the birth; transport in the case of an obstetric emergency; and identification of compatible blood donors in case of emergency [4]. A meta-analysis by *Soubeiga D*, *et al*. [6] has reported that BPCR interventions, with adequate population coverage, showed a significant change in neonatal mortality, but a non-significant reduction of maternal mortality.

A set of indices has been established by JHPIEGO (an affiliate of Johns Hopkins University, USA) to measure the BPCR at six different levels: the individual woman, her family (husband/partner), the community, the healthcare provider, the health facility, and the policy environment [4]. Pregnant and recently delivered women are the key target population for the individual level assessment of BPCR. While recently delivered women can provide a full range of information for BPCR assessment, not all pregnant women, especially those in the early stage of pregnancy will do so. [4].

India has made a significant progress in reducing the number of maternal deaths in the last two decades. From 1990 to 2013, the Maternal Mortality Ratio (MMR) in India has declined from 600 to 167 per 100,000 live births [7]. The highest decline was from 2004–06, which coincides with the period after the launch of National Rural Health Mission, and the numerous initiatives taken under this flagship scheme, including the *Janani Suraksha Yojana* (JSY) which has resulted in a surge in the institutional deliveries [1]. JSY is a 100% centrally sponsored scheme launched in 2005 by modifying the National Maternity Benefit Scheme. The aim of this program is to reduce the maternal and neonatal mortality by promoting institutional delivery among poor pregnant women [8]. It provides a graded scale of cash assistance (from 600–1,400 Indian rupees) based on the categorization of states and place of residence (rural/urban). Based on the institutional delivery rates, states were categorized as ‘low’ (<25%) and ‘high’ (>25%) performing [8]. In high-performing states, a woman >19-year-old belonging to below poverty line/scheduled caste/tribe delivering in a public health institution or JSY accredited private hospital is eligible for the cash assistance. The assistance is limited to the first two live births only. However, in low-performing states, all the pregnant women are eligible. [8].

At the grassroots level, Auxiliary Nurse Midwife (ANM), for every 5,000 rural populations, renders reproductive and child health services [9]. A community health volunteer called Accredited Social Health Activist (ASHA), for every 1000 rural populations, has been engaged under the National Rural Health Mission (NRHM). ASHAs facilitate the ANM (by creating awareness and community mobilization) to render maternal and child health services and establish a link between the community and the healthcare system [10].

Karnataka, a southern state of India, is categorized as a high-performing state under National Rural Health Mission. The MMR in the state is 144/100,000 live births with an annual decline of 6.8% (2011–12) [1]. *Dakshina Kannada* is a coastal district of Karnataka and is the second most developed district in the state with a human development index of 0.687 (Human Development Report 2014) [11]. According to District Level Household Survey (DLHS) -IV 2012–13, 98.3% of the rural pregnant women in *Dakshina Kannada* had an institutional delivery. However, a substantial proportion of women in the district experience complications during pregnancy (58.3%), childbirth (45.7%), and postnatal (17.4%) period [12]. There is a paucity of data on BPCR and the factors associated with it in this district. Pregnancy and childbirth are not merely biological phenomena. Woman’s age, literacy, socioeconomic status, ethnic background, religion, and culture have a significant influence on the experiences and outcome of pregnancy [13]. Exploring the key factors associated with BPCR would be of great use in evidence-based fine-tuning of ongoing maternal and child health interventions to minimize the complications and avert maternal deaths. Therefore, this study aimed to assess the BPCR practices and the factors associated with it among the beneficiaries of two rural Primary Health Centers (PHCs) of *Dakshina Kannada* district, Karnataka, India.

## Methods

### Study setting

This study was conducted in two rural PHCs of *Dakshina Kannada* district, Karnataka, India. The district lies between 12 57' and 13 50’ North Latitude and 74 and 75 50’ East longitude on the western coast of India. Total population of the district is 2,089,649, spread over a geographical area of 4,859 square kilometers. Average literacy rate of the district is 88.57% (rural-85.33% and urban-92.12%). The district is divided into five *talukas*. *Taluka* is an area of the land with a city or town that serves as its administrative centre and a number of villages. The public health infrastructure of the district consists of one district hospital, eight community health centers, four first referral units, and sixty-five rural PHCs.

The total number of pregnant women in the district was 28,690 from Apr 2013-Mar 14 (Source: District Health Office, Dakshina Kannada). Being a high-performing district, a woman in *Dakshina Kannada* district belonging to below poverty line family and scheduled caste/tribe category is eligible for JSY benefits for her first two deliveries. In this district, 1072 ASHAs have been appointed and trained by 2011. ASHAs have been imparted an induction training in the beginning for 23 days spread in five rounds over a period of 12 months and followed by periodic re-training for about two days once in two months.

The key roles of ASHA are to be the first port of call for any health and health-related demands of women and children, to counsel women about BPCR, importance of safe delivery, breastfeeding and complementary feeding, immunization, contraception, etc. ASHAs also mobilize and facilitate the women in accessing available services at the *Anganwadi*/sub-centre/PHC [10]. All these services are rendered free of cost to the beneficiaries, and the ASHA gets performance-based monetary incentives for all her activities.

### Study design and sample

A facility-based cross-sectional study was conducted from June-September, 2013. Based on the reported individual level BPCR index (average of seven indicators assessing the knowledge of key danger signs, knowledge of community resources, service use, and planning actions) of 47.5% (~47%) in a study from middle part of India [14], this study required a sample size of 196 for estimating the expected proportion with 7% absolute precision and 95% confidence [15]. Anticipating a non-response rate of 10%, it was decided to approach 217 eligible participants. Keeping the resource constraints in mind, it was decided to include two rural PHCs of different *talukas* in this study. Two of the five *talukas* (*Mangaluru and Bantwal*) in the district were selected by simple random sampling. One rural PHC was randomly selected from each of the *talukas*. Selected rural PHCs cater to the needs of nearby villages and cover a population of 53,774.

#### Inclusion and exclusion criteria

Pregnant women with >28 weeks of gestation (considering that woman should have followed all the components of BPCR by this time) and recently delivered (in the last 6 months to minimize the recall bias) women, irrespective of the pregnancy outcome, attending the selected PHCs were included. Pregnant women in active labor, mentally or physically incapable of exit interview or those not willing to participate were excluded.

### Data collection and analysis

A semi-structured pretested interview schedule was used to collect the relevant data. In the study district, pregnant and recently delivered women come to PHC for routine check-up on Tuesday and Thursday, respectively. All of them bring the ‘mother-child protection card’. The participants’ cards were marked to exclude them in the subsequent visits. Four medical interns were trained to conduct exit interview of the eligible study participants. Information regarding socio-demographic profile, obstetric details, the primary decision maker in the family (regarding planning and place of healthcare), knowledge of the key danger signs and BPCR practice, was elicited. Medical interns were trained to extract the information by explaining the participants in local language *Kannada*. The study participants were given ample time to ask/clarify doubts if they could not understand. At the end of the interview, all the responses of a participant were read again to re-confirm her response. Anonymity of the study participants was maintained to ensure confidentiality and also to enhance the participation rate.

Interview schedule included following details

Socio-demographic profile: age in years, religion, literacy status, working status, economic status, husband’s literacy and working status, household size (number of people who occupy a housing unit) and primary decision maker in the family regarding the timing and place of seeking healthcare during pregnancy. Obstetric details: gravidity, parity, number of antenatal care (ANC) visits done, preferred source of ANC or childbirth, awareness of JSY benefits. Knowledge of key danger signs of pregnancy (vaginal bleeding, swollen hands/face, blurred vision and convulsion), childbirth (severe vaginal bleeding, prolonged labour, i.e., >12hours, convulsion and retained placenta) and postpartum (severe vaginal bleeding, foul-smelling vaginal discharge, and high fever). All these danger signs are depicted in the mother-child protection card which is given to all the pregnant women by ANM while imparting essential obstetric care. ANM or ASHA is expected to sensitize the pregnant woman about the key danger signs with the help of mother-child protection card. All the study participants were asked to enlist these key dangers of pregnancy, childbirth and postpartum without any probing. BPCR information and practice: identified the place of delivery, saved money to pay for expenses, identified the mode of transport to the place of childbirth, identified a birth companion and arranged a blood donor if the need arises.

Literacy status of the study participant was categorized as literate (if one can read and write with understanding in any language) or illiterate (can neither read nor write /can read but cannot write in any language) and literacy level was the highest level of education completed. (Census India 2011) [16]. Working status of the woman was categorized as employed (engaged in economically productive work) or unemployed. In this study, the type of ration card possessed by the woman was taken as a proxy indicator of her economic status, i.e., red/green and blue colored cards for below and above poverty line families, respectively (BPL and APL).

A woman who recalled at least two key danger signs in each of the three phases, i.e., pregnancy, childbirth, and postpartum (total six danger signs) was considered as knowledgeable on key danger signs. A woman who received information about all the five key elements of BPCR was defined as adequately informed. Optimal BPCR was defined as following at least three out of five key components of BPCR.

Data were analyzed using Statistical Package for the Social Sciences (SPSS) for Windows, Version 16.0. Chicago, SPSS Inc. Results were expressed as frequencies and proportions for categorical variables and mean and standard deviations for continuous variables. Chi-square test was applied to assess the differences in BPCR across various study variables. A two-sided p-value of <0.05 was considered as statistically significant. Multivariate logistic regression was applied to examine the simultaneous impact of the study variables on optimal BPCR practice. Proportion, Odds ratio (OR), and adjusted Odds ratio (adj OR) with 95% confidence intervals for the optimal BPCR practice were the key outcome measures.

## Ethical approval

Institutional review board and ethics committee of Yenepoya University, Mangaluru, India approved the study protocol (YUEC119/2013 dated 1^st^ June 2013). Permission was also obtained from the medical officers of the study PHCs. Informed written consent was taken from all the study participants for voluntary participation in local language, *Kannada*. If the woman was illiterate then the details of the study were explained in the presence of a witness and left thumb impression of the participant and the signature of the witness was taken on the consent form.

## Results

A total of 217 eligible (pregnant with >28 weeks of gestation or delivered in last 6 months) women were approached. Of these, 184 (60 pregnant and 124 recently delivered) women participated in this study (response rate: 84.8%). Table 1 shows the key socio-demographic and obstetric parameters of the study participants. Most of them (n = 146, 79.4%) belonged to the age group of 21–30 years and their mean (±SD) age was 26.9 (±3.9) years [Table 1]. A majority of them were primigravida/primiparas (n = 108, 58.7%) and homemakers (n = 145, 78.8%) by occupation. The median household size was 5 (range: 2–15). Observed difference in the literacy levels between women (95.7%) and their husbands (96.7%) was not statistically significant (χ^2^ = 0.251, p = 0.616). A majority of them were from economically BPL family (n = 104, 56.5%) and public health sector was the source of ANC/delivery for 52.7% of the women. Preference to public healthcare facility for ANC/delivery was significantly high among women from BPL families (61.5% vs. 41.3%, p = 0.006) and with low literacy level, i.e., up to primary school (66.7% vs. 49%, p = 0.049).

**Table 1:** Socio demographic and obstetric parameters of pregnant (≥28 weeks of gestation) and recently (within 6 months) delivered women attending two rural primary health centers, *Dakshina Kannada* district, Karnataka, India, June-September 2013 (n = 184).

Study variable	n	%
**Age (years)**		
18–20	7	3.8
21–25	64	34.8
26–30	82	44.6
31–35	24	13.0
>35	7	3.8
**Religion**		
Hindu	113	61.4
Islam/Christian	71	38.6
**Gravidity/Parity**		
Primgravid/ Primipara	108	58.7
Multigravid/ Multipara	76	41.3
**Household size**		
≤5	114	62.0
>5	70	38.0
**Education status**		
Illiterate	8	4.3
Up to secondary school	137	74.5
High school and above	39	21.2
**Working status**		
Unemployed	145	78.8
Employed	39	21.2
**Husband’s education status**		
Illiterate	6	3.3
Up to secondary school	135	74.4
High school and above	43	23.3
**Husband’s occupation**		
Skilled or professional	90	48.9
Unskilled	94	51.1
**Economic status**		
Above poverty line	80	43.5
Below poverty line	104	56.5
**Preferred source of antenatal care /delivery**		
Public health sector	97	52.7
Private health sector	87	47.3
**Number of Antenatal care visits done**		
<4	40	21.7
≥4	144	78.3
**Aware of *Janani Suraksha Yojana***		
No	89	48.4
Yes	95	51.6
**Key danger signs**		
Aware of at least one danger sign	147	79.9
Aware of at least six danger signs	99	53.8
None	37	20.1
**Decision maker in seeking healthcare**		
Self	19	10.3
Husband/others	165	89.7
**Companion for antenatal care**		
None	26	14.1
Husband/mother/mother-in-law	158	85.9
**Adequately informed about BPCR**^#^		
Yes	114	62.0
No	70	38.0
**Discussed BPCR**^#^ **with family members**		
Yes	117	63.6
No	67	36.4

^**#**^ BPCR: birth preparedness and complication readiness

Although 78.3% (n = 144) of the women had ≥4 ANC visits during their pregnancy, only 47.9% (n = 69) of them were aware of JSY. Only 10.3% of the women were the primary decision maker in their family regarding when and where to seek the healthcare during the pregnancy. As many as 158 (85.9%) women were accompanied by her husband/mother/mother-in-law for ANC visits. Almost 80% of the women were aware of at least one danger sign. As much as 53.8% of the women were knowledgeable on key danger signs (could recall six key danger signs three phases i.e. pregnancy, childbirth and postpartum). Almost two-thirds of them (n = 114, 62%) were adequately informed about BPCR by a doctor/ANM/ASHA and discussed BPCR with their family members (n = 117, 63.6%). Optimal BPCR practice was observed in 79.3% (95% CI: 73.5–85.2%) of the women.

Following percentage of the women practiced the key components of BPCR: identified the place of delivery (n = 184, 100%), saved money to pay for expenses (n = 96, 52.2%), identified the mode of transport to the place of childbirth (n = 132, 71.7%), identified a birth companion (n = 167, 90.8%) and arranged a blood donor if the need arises (n = 29, 15.8%).

Awareness of minimum six key danger signs was significantly higher (p = <0.001) among those who received adequate information on BPCR than those who did not (64.9% vs. 35.7%). Woman’s discussion with family members regarding BPCR was significantly higher (p = <0.001) among those who received adequate BPCR information than those who did not (83.3% vs. 31.4%).

Optimal BPCR practice did not differ significantly (p = 0.535) between pregnant (n = 46, 76.7%) and recently delivered (n = 100, 80.6%) women. Tables 2 and 3 showed that the following factors were associated with the higher odds of optimal BPCR practice: economic status of APL (OR = 3.65; 95%CI:1.6–8.5, p = 0.002), preference to private health sector for ANC/delivery (OR = 3.13; 95%CI: 1.42–6.9, p = 0.004), completing ≥4 ANC visits (OR = 3.5; 95%CI: 1.5–6.9, p = 0.003), awareness of at least six (OR = 5.18; 95%CI: 2.28–11.76, p = <0.001) danger signs of pregnancy, intra and postpartum, receiving adequate information about BPCR (OR = 2.13; 95%CI: 1.04–4.4, p = 0.038) and discussing BPCR with family members (OR = 3.56; 95%CI: 1.69–7.5, p = 0.001).

**Table 2:** Association between birth preparedness and complication readiness (BPCR), and socio-demographic and obstetric parameters of women attending two rural primary health centers, *Dakshina Kannada* district, Karnataka, India, June-September 2013 (n = 184).

Study variable	BPCR					χ^2^	p
	Suboptimal (n = 38)		Optimal (n = 146)^#^		Total		
	n	%	n	%			
**Age (years)**							
≤26	24	25.3	71	74.7	95	2.548	0.11
>26	14	15.7	75	84.3	89		
**Religion**							
Hindu	23	20.4	90	79.6	113	0.016	0.9
Islam/Christian	15	21.1	56	78.9	71		
**Gravidity/Parity**							
Primgravida/ Primipara	20	18.5	88	81.5	108	0.726	0.394
Multigravid/ Multipara	18	23.7	58	76.3	76		
**Household size**							
≤5	25	21.9	89	78.1	114	0.299	0.585
>5	13	18.6	57	81.4	70		
**Education status**							
Illiterate/up to secondary school	28	19.3	117	80.7	145	0.752	0.386
High school and above	10	25.6	29	74.4	39		
**Working status**							
Unemployed	29	20	116	80	145	0.178	0.673
Employed	9	23.1	30	76.9	39		
**Husband’s education status**							
Illiterate/up to secondary school	31	22	110	78	141	0.655	0.418
High school and above	7	16.3	36	83.7	43		
**Husband’s occupation**							
Skilled or professional	17	18.9	73	81.1	90	0.334	0.563
Unskilled	21	22.3	73	77.7	94		
**Economic status**							
Above poverty line	8	10.0	72	90.0	80	9.801	**0.002**^§^
Below poverty line	30	28.8	74	71.2	104		
**Preferred source of antenatal care /delivery**							
Public health sector	28	28.9	69	71.1	97	8.446	**0.004**^§^
Private health sector	10	11.5	77	88.5	87		
**Number of antenatal care visits**							
<4	15	37.5	25	62.5	40	8.85	**0.003**^§^
≥4	23	16	121	84	144		
**Aware of *Janani Suraksha Yojana***							
No	20	22.5	69	77.5	89	0.349	0.555
Yes	18	18.9	77	81.1	95		
**Knowledge of key danger signs**							
Aware of at least six danger signs	9	9.1	90	90.9	99	17.48	**<0.001**^§^
Aware of <6 danger signs/unaware	29	34.1	56	65.9	85		
**Decision maker in seeking healthcare**							
Self	5	26.3	14	73.7	19	0.415	0.52
Husband/others	33	20	132	80	165		
**Companion for antenatal care**							
None	7	26.9	19	73.1	26	0.727	0.394
Husband/mother/mother-in-law	31	19.6	127	80.4	158		
**Adequately informed about BPCR**							
Yes	18	15.8	96	84.2	114	4.32	**0.038**^§^
No	20	28.6	50	71.4	70		
**Discussed BPCR with family members**							
Yes	15	12.8	102	87.2	117	12.03	**0.001**^§^
No	23	34.3	44	65.7	67		

^#^Any 3 of 5 steps: identified a health facility, arranged for transport, identified blood donor, identified a birth companion and saved money for emergency;

^§^Significant (p<0.05)

**Table 3:** Crude and adjusted Odds ratios (OR) for the optimal birth preparedness and complication readiness (BPCR) among women attending two rural primary health centers, *Dakshina Kannada* district, Karnataka, India, June-September 2013 (n = 184).

Variable for optimal BPCR^#^	OR	95% CI	Adjusted OR^†^	95% CI
**Age (years)**				
≤26	1		1	
>26	1.811	0.87–3.78	2.97	**1.15–7.7**^§^
**Religion**				
Hindu	0.954	0.46–1.98	0.7	0.24–2.03
Islam/Christian	1		1	
**Gravidity/Parity**				
Primgravid/ Primipara	0.73	0.36–1.5	1.8	0.64–4.8
Multigravid/ Multipara	1		1	
**Household size**				
>5	1.23	0.58–2.6	1.01	0.4–2.56
≤5	1		1	
**Education status**				
Illiterate/up to secondary school	1		1	
High school and above	1.44	0.63–3.3	0.8	0.26–2.4
**Working status**				
Unemployed	1		1	
Employed	0.833	0.36–1.95	0.87	0.3–2.55
**Husband’s education status**				
Illiterate/up to secondary school	1.45	0.59–3.57	0.5	0.13–1.83
High school and above	1		1	
**Husband’s occupation**				
Skilled or professional	0.81	0.395–1.66	1.4	0.5–3.87
Unskilled	1		1	
**Economic status**				
Above poverty line	3.65	**1.6–8.5**^§^	4.3	**1.12–16.5**^§^
Below poverty line	1		1	
**Preferred source of antenatal care /delivery**				
Private health sector	3.125	**1.42–6.9**^§^	2.9	**1.1–8.01**^§^
Public health sector	1		1	
**Antenatal care visits done**				
<4	1		1	
≥4	3.5	**1.5–6.9**^§^	2.4	0.83–6.96
**Aware of *Janani Suraksha Yojana***				
No	1		1	
Yes	1.24	0.61–2.5	1.6	0.55–4.54
**Knowledge of key danger signs**				
Aware of at least six danger signs	5.179	**2.28–11.8**^§^	3.98	**1.4–11.1**^§^
Aware of <6 danger signs/unaware	1		1	
**Decision maker in seeking healthcare**				
Self	0.7	0.24–2.08	3.04	0.72–12.8
Husband/others	1		1	
**Companion for antenatal care**				
None	1			
Husband/mother/mother-in-law	1.51	0.58–3.91	1.04	0.3–3.6
**Adequately informed about BPCR**				
Yes	2.13	**1.04–4.4**^§^	1.37	0.43–4.4
No	1		1	
**Discussed BPCR with family members**				
Yes	3.56	**1.69–7.5**^§^	3.4	**1.1–10.4**^§^
No	1		1	

OR = Odds ratio; CI = Confidence Interval;

^#^Any 3 of 5 steps: identified a health facility, arranged for transport, identified blood donor, identified a birth companion and saved money for emergency;

^§^Significant (p<0.05);

^†^Adjusted for all the independent variables indicated in the table

On applying logistic regression, woman’s age >26 years (adj OR = 2.97; 95%CI: 1.15–7.7), economic status of APL (adj OR = 4.3; 95%CI: 1.12–16.5), awareness of minimum six key danger signs of pregnancy, childbirth and postpartum (adj OR = 3.98; 95%CI: 1.4–11.1), preference to private health sector for ANC/delivery (adj OR = 2.9; 95%CI: 1.1–8.01), and woman’s discussion about the BPCR with her family members (adj OR = 3.4; 95%CI: 1.1–10.4) remained as the significant factors of optimal BPCR practice in the study population [Table 3]. The applied regression model could explain a variance of 38.2% in the BPCR which was not high but within acceptable limits.

## Discussion

Nearly eight out of every ten women (79.3%) fulfilled the operational criteria of optimal BPCR practice. The higher odds of optimal BPCR practice were observed for the woman’s age (>26 years), economic status of APL, knowledge of at least six key danger signs of pregnancy, childbirth and postpartum, preference to private health sector for ANC/delivery and woman’s discussion about BPCR with family members.

The observed BPCR practice in this study is higher than the other Indian studies conducted in Madhya Pradesh (47.8%, n = 312) [17], Delhi (41%, n = 417) [18] and West Bengal (49.4%, n = 240 and 34.5%, n = 355) [19,20]. A wide range of BPCR (16.5–65%) has been reported from many countries like Ethiopia (16.5–29.9%) [21–24], Uganda (35%) [25], Tanzania (58.2%) [26], Nepal (32–65%) [27,28]. These variations could be attributed to different levels of female literacy and empowerment, spouse’s education and occupation, knowledge of key danger signs, preference to institutional delivery and methodological differences in BPCR assessment. Relatively high BPCR in the present study could be due to high female literacy, better knowledge of danger signs, higher service utilization, and a higher proportion of institutional deliveries in the study district [12].

The significant influence of woman’s literacy level on BPCR practice is highlighted by many studies from India and African countries [17,29–31]. However, in our study and a study by *Timša L*, *et al* [32], woman’s education level did not show a significant association with BPCR. This could be attributed to overall high (95.6%) literacy rate in this study population. Many studies have reported that optimal BPCR practice is associated with woman and spouse’s working status, religion, multiparity and adequate number of ANC visits [17–22]. But, such associations were not noted in this study. Overall, high utilization of available maternal healthcare services and preference to institutional delivery is the possible explanation [12].

This study has highlighted the direct positive effect of the knowledge of the key danger signs on BPCR. Other studies [18,22] have also attributed unsatisfactory BPCR practice to poor knowledge of the key danger signs. An adequate knowledge of danger signs aids in early recognition of potentially life-threatening complications and may avert the unnecessary delay in seeking healthcare [4]. ANM and ASHAs should be encouraged to educate the expectant mother and her family members about the danger signs during ANC.

In rural parts of the study district, 39.7% of the deliveries occur in public health institutions [12]. In our study, most of the women who preferred public health sector were from BPL family. Both poverty and preference to public healthcare facility for ANC/delivery were the independent factors of suboptimal BPCR practice. A low-level of awareness of available schemes (only 51.6% were aware of JSY) may be the possible explanation. Awareness campaigns should be conducted to explain the various monetary and non-monetary benefits of JSY.

Postpartum hemorrhage and anemia are the most common direct and indirect causes of maternal mortality in India, respectively [33]. In this context, identification of a compatible blood donor and availability in case of an emergency may be life-saving especially in facilities where blood is scarce. However, similar to this study, others have reported that very few pregnant or recently delivered women identified the blood donor [20,21,34].

In rural parts of *Dakshina Kannada* district, out-of-pocket expenditure per institutional delivery in a public health facility is 2,910 Indian rupees [12]. This, to a larger extent, is addressed by JSY (including free transport facility). However, the monetary benefits are sanctioned only after the delivery. Therefore, saving money to pay for the delivery expenses is important. In this study, such decisions were taken by men/others in the family. Considering the patriarchal norms of the Indian society, this is not an unexpected finding. ANM and ASHA should involve the family members, especially the spouse, while educating woman about these factors during ANC. A special emphasis on young and economically poor pregnant women is needed. Studies have shown that involving the members, especially the spouse, ensures implementation and sustainability of BPCR [35–37].

In addition, community, health system, and provider related factors have a significant influence on BPCR and need to be explored. Furthermore, perceived susceptibility, severity, and benefits of the services by woman also affect the level of BPCR [38]. Existing evidence suggests that culture has a strong influence on women’s use of available healthcare services [39–41]. WHO recommends that culture and its dynamism need to be recognized, anticipated and incorporated into maternal healthcare services [42]. Qualitative research and encouraging community participation while designing the intervention would be of great use to explore and address the cultural factors [42].

With due consideration of a satisfactory level of optimum BPCR practice and better utilization of available maternal health services, the focus can now shift to the quality of services being rendered at the grassroots level. Imparting adequate knowledge about key danger signs and stressing the importance of BPCR are needed. In this context, the role of ANM and ASHA is critical. Involving the family members while discussing key danger signs and BPCR will further enhance the implementation.

### Limitations

Facility based study sample may not exactly represent the pregnant and recently delivered women in the community and a relatively small sample was studied. Hence, external validity of the findings is questionable. Women with abortion or stillbirth (overall prevalence in the district: 4.7%) [12] did not come for follow-up to PHCs during the study period. Therefore, we could not study their association with BPCR. The authors did not consider knowledge of neonatal danger signs while assessing BPCR. Owing to cross-sectional study design, the associations observed in this study may not imply causality.

## Conclusion

In this study population, BPCR practice was better than other studies reported from India. Optimal BPCR practice was observed among women aged >26 years, economically above the poverty line, had knowledge of at least six key danger signs, preferred private health sector for ANC/delivery, and discussed BPCR with family members. Healthcare workers at the grassroots (ANM and ASHA) should be encouraged to involve women’s family members while explaining BPCR and key danger signs with a special emphasis on young (<26 years) and economically poor women. Ensuring a reinforcing discussion between woman and her family members may further enhance the BPCR practice.

## Supporting information

S1 File Data sheet. (XLSX)

S2 File STROBE checklist. (DOCX)
